# Neuropathy caused by B12 deficiency in a patient with ileal tuberculosis: A case report

**DOI:** 10.1186/1752-1947-2-90

**Published:** 2008-03-21

**Authors:** Taraneh Dormohammadi Toosi, Farhad Shahi, Ali Afshari, Nader Roushan, Marjan Kermanshahi

**Affiliations:** 1 Imam Khomeini Hospital, Tehran University of Medical Sciences, Keshavarz Blvd., Tehran, Iran

## Abstract

**Introduction:**

Vitamin B12 deficiency can result in macrocytic anemia. Neurologic abnormalities of B12 deficiency include sensory deficits, loss of deep tendon reflexes, movement disorders, neuropsychiatric changes and seizures. Segmental involvement of the distal ileum, such as in tuberculosis, can cause vitamin B12 deficiency. To our knowledge, macrocytic anemia with unusual manifestations such as brain atrophy and seizures due to intestinal tuberculosis has not been reported in the literature.

**Case presentation:**

A 14-year-old girl presented with complaints of paraplegia, ataxia, fever and fatigue that had started a few months earlier and which had been getting worse in the last three weeks. Her laboratory results were indicative of macrocytic anemia with a serum B12 level <100 (normal, 160–970) pg/ml and hypersegmented neutrophils. Her MRI findings showed brain atrophy. Her fever workup eventually led to the diagnosis of tuberculosis which was documented by bone marrow aspiration smear & culture. A small bowel series showed that tuberculosis had typically involved the terminal ileum which had resulted in vitamin B12 deficiency. She was treated for vitamin B12 deficiency and tuberculosis. Her fever ceased and her hemoglobin level returned to normal. At present, she can eat, write, and speak normally as well as walk and ride a bicycle.

**Conclusion:**

Vitamin B12 deficiency should be considered in patients with neurologic features such as paresthesia, sensory deficits, urinary incontinence, dysarthria, and ataxia. The underlying cause of B12 deficiency should be determined and treated to obviate the patients' need for long term vitamin B12 therapy.

## Introduction

Vitamin B12 deficiency leads to delayed DNA synthesis in rapidly growing hematopoietic cells, and can result in macrocytic anemia. Neurologic abnormalities of B12 deficiency include paresthesia, sensory deficits, loss of deep tendon reflexes, movement disorders, developmental regression, dementia and neuropsychiatric changes [1]. Magnetic resonance imaging (MRI) has been able to demonstrate brain atrophy and delayed myelination in these cases [2]. B12 deficiency may also cause seizures [3,4]. Possible causes of vitamin B12 deficiency in childhood include decreased intake, abnormal absorption and defects in vitamin B12 transport and metabolism. Segmental involvement of the distal ileum, such as in tuberculosis, can cause macrocytic anemia [5,6]. To our knowledge, macrocytic anemia with unusual neurologic manifestations due to intestinal tuberculosis has not been reported previously in the literature.

## Case presentation

A 14-year-old girl presented with complaints of fatigue, inability to walk, urinary incontinence, dysarthria, and ataxia that had worsened in the last three weeks. She also had fever, weight loss and decreased appetite. In her past medical history she had been treated for seizures with valproic acid (15 mg/kg/day) for three years. Her seizures were documented by EEG findings.

On physical examination, her blood pressure was 110/60 mmHg and she had a pulse rate of 88/min, a respiratory rate of 14/min and oral temperature of 38.6°C. Her growth and development were normal, reaching menarche at the age of 13, followed by regular cycles until two months before admission. Her weight was 48 kg and her height was 154 cm. She had received all vaccinations since birth according to the national vaccination plan. Her family history was negative for any hereditary or metabolic disorders.

Her consciousness level was normal and she had no neck stiffness. In spite of being awake, she could not communicate with anyone and only used indistinct words. The pupils were normal in size and equally reactive to light. Her sclera were pale.

On neurological examination, her upper limbs were mildly spastic, and her muscle strength was 2–3/5 as far as she could cooperate. Her upper limb reflexes, including the biceps, brachioradialis, and triceps, and also her sensory examinations were normal.

Her lower limbs, however, were flaccid and atrophic; the muscle strength was 0/5 and the patellar and Achilles reflexes were absent. She showed an extensor plantar reflex. Her lower limb sensory examination showed lost senses of light touch, pain, temperature, vibration and joint position. This may have been unreliable due to her overall condition.

As she was not able to stand or walk, gait and cerebellar examinations were not performed. We also found a deep infected 5 × 4 cm bedsore in her sacral area, as a result of being bedridden for more than three weeks. Her abdominal, chest and heart examinations were normal. Her hemoglobin was 8.3 gr/dl (See Additional file 1: Table 1 for the CBC result). All other blood tests were normal (See Additional file 2: Table 2 for other blood tests). Her cerebrospinal fluid examination and chest X-ray were also normal.

B12 deficiency was documented by serum vitamin B12 level <100 (normal, 160–970) pg/ml and peripheral blood smear that showed hypersegmented neutrophils (6 and 7 segments).

**Figure 1:**
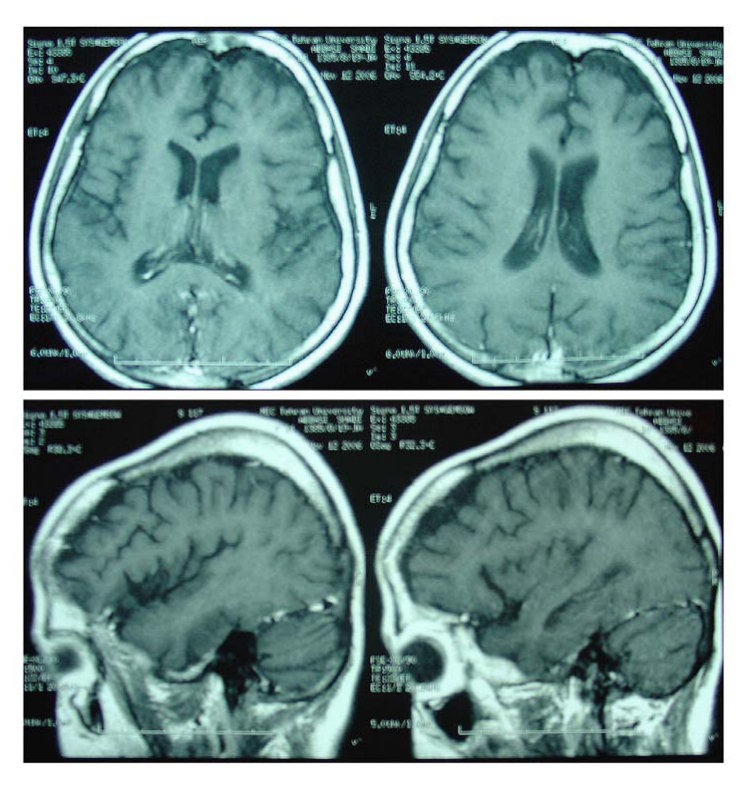
Brain MRI shows senile dilatation in the CSF space and sulci of brain hemispheres that is compatible with mild atrophic changes.

EEG findings included some sharp activity that could indicate epileptogenic activity. The MRI demonstrated senile dilatation in the CSF space and sulci of both hemispheres; a finding compatible with mild atrophic changes (Fig. 1).

EMG/NCV showed prolonged distal latency of motor nerve, decreased amplitudes of compound muscle action potential, decreased conduction velocity, and increased F wave latencies. Sensory nerve action potentials were not detected. All above finding are compatible with mixed sensorimotor polyneuropathy.

Treatment was begun with vitamin B12 (1 mg/IV/day) and folic acid (5 mg/oral/day). After a few weeks, her symptoms improved and she was able to speak and eat. Her upper limb mobility improved and she began to communicate with us. Her reticulocyte count also increased dramatically from 0.9% to 7.1% (on the 7^th ^day of treatment) indicating a favorable treatment response. However, she was febrile in spite of being treated with vancomycin (1 gr/BID) and imipenem (500 mg/QID) which were chosen based on the infected bed sore cultures and antibiograms. After one month, her hemoglobin slightly rose and remained in the range of 8–9 gr/dl. Although her serial blood cultures were negative, she remained febrile, and we suspected an unusual source of infection that did not respond to broad spectrum antibiotics. Therefore, a bone marrow aspiration and biopsy was performed three weeks after initiating the treatment, and the specimen was examined for unusual germs such as tuberculosis and brucellosis.

**Figure 2:**
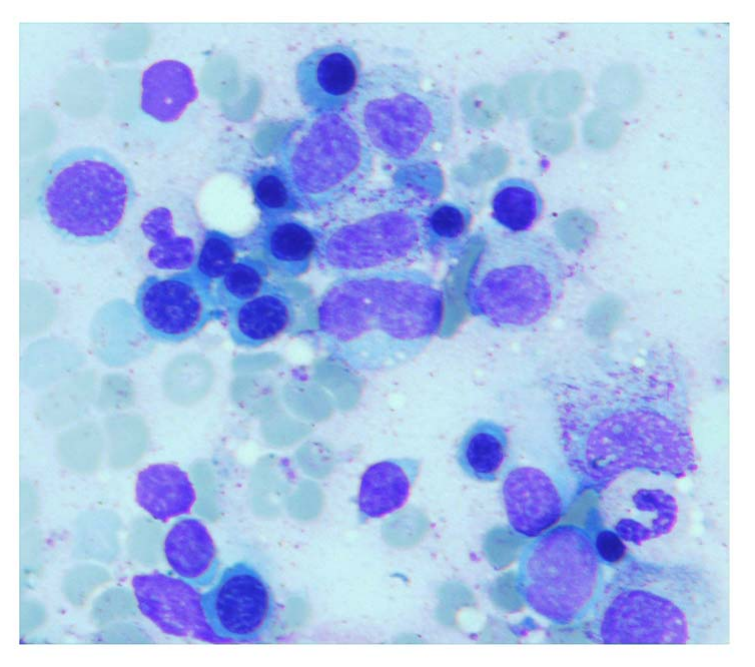
Bone marrow examination showed some degree of megaloblastic changes, giant metamyelocytes and nuclei/cytoplasm dissociation (Bone marrow aspiration and biopsy were taken 3 weeks after commencement of B12 treatment).

Bone marrow examination (Fig. 2) showed some degree of megaloblastic changes, giant metamyelocytes and nuclei/cytoplasm dissociation. The laboratory report showed there were acid fast bacilli in her bone marrow aspiration smear. Therefore, we started a six month antituberculosis treatment with isoniazid 5 mg/kg/day, rifampin 10 mg/kg/day, ethambutol 15 mg/kg/day, and pyrazinamide 20 mg/kg/day.

**Figure 3:**
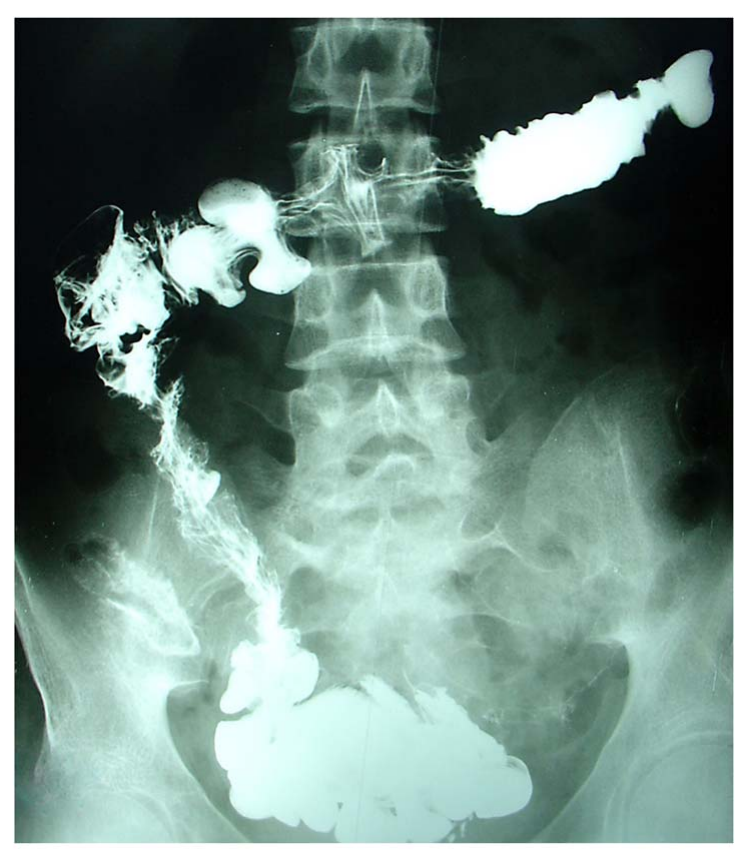
Small bowel series (small bowel transit) shows narrowing and irregularity of the terminal ileum and ulceration in its wall, highly suspicious for tuberculosis.

This treatment resulted in the termination of fever on the second day, further elevation of the hemoglobin level and improvements in her general health. Her upper-limb force became normal and she was able to eat by herself. Her lower-limb force improved and the plantar reflex was now flexor. To find the cause of megaloblastic anemia, we reassessed the case. Valproic acid was not the cause, she was not a vegetarian, and she had no sign or symptoms of malabsorbtion or malnutrition. Her growth and development were normal. We concluded that her vitamin B12 deficiency may be of a gastrointestinal origin. As the Schilling test was not available at our center, we conducted a small bowel series by barium examination and the results showed terminal ileum narrowing, irregularity and mucosal ulceration; all highly indicative of tuberculosis (Fig. 3).

Colonoscopy showed a normal colon but a severely strictured and edematous ileocecal valve, obstructing the path to the terminal ileum for the endoscopic tube.

We concluded that M. tuberculosis had caused the terminal ileum disease and possibly vitamin B12 deficiency in our patient. At present, our patient has been treated for vitamin B12 deficiency and M. tuberculosis. After being treated with anti-tuberculosis drugs her fever terminated and her hemoglobin level returned to normal. Currently, 10 months after admission, she can eat, write, and speak normally as well as walk and ride a bicycle, but she has some degree of foot drop. Her latest tests showed a hemoglobin level of 13.7 gr/dl, hematocrit 39.4, and her MCV was 89.3 fl. The small intestine barium examination at the completion of the anti-tuberculosis treatment showed less narrowing and irregularity, which further confirmed the diagnosis.

## Conclusion

Vitamin B12 deficiency causes neurologic abnormalities such as paresthesia, movement disorders, developmental regression and neuropsychiatric changes [1]. Our patient suffered from fatigue, inability to walk, urinary incontinence, dysarthria, and ataxia. These manifestations could be attributed to B12 deficiency. In one study on 50 patients with vitamin B12 deficiency and megaloblastic anaemia, the commonest finding was peripheral neuropathy, but subacute combined degeneration of the cord was uncommon. About a quarter of these patients had either cognitive impairment or an affective disorder, but a third had no detectable nervous system involvement [7].

The mechanism of neurological effects in cobalamin deficiency is not fully elucidated. Impaired methionine synthesis may lead to depletion of S-adenosylmethionine which is required for the synthesis of myelin phospholipids. The second hypothesis is that a deficit of succinyl-CoA leads to the generation of odd chained fatty acids which may get incorporated into the myelin and cause the neurological syndrome of Vitamin B 12 deficiency [6].

As in our patient, brain atrophy and delayed myelination can be observed on MRI [2]. Vitamin B12 deficiency may also cause seizures. The exact mechanism of epileptogenesis in cobalamin deficiency is not clear. It is likely that cerebral neurons with destroyed myelin sheaths are more susceptible to the excitatory effects of glutamate. Serum B12 levels should be checked, especially in patients who present with other known neuropsychiatric features of vitamin B12 deficiency [3]. Our patient's seizures may have been due to B12 deficiency. We believe she can taper and end the antiepileptic drugs under close EEG and continual symptom monitoring, after complete recovery [4]. She also suffered from fever, especially at night, weight loss and reduced appetite. Fever is an unusual finding in B12 deficiency unless it is accompanied with another disease [8]. Her fever workup showed there were acid fast bacilli in her bone marrow aspiration smear, further confirmed by a positive culture after 48 days.

After treatment with anti-tuberculosis drugs, her fever terminated, her hemoglobin level elevated and her general condition improved. This response to therapy was a good indication confirming the involvement of tuberculosis.

In the workup for megaloblastic anemia, we suspected valproic acid as a cause [9]. Antiepileptic medications, such as Valproic acid can cause macrocytosis, mainly by reducing serum folic acid levels [10]; this was not the case in our patient (serum folic acid = 17 ng/ml). It has also been reported that long term anticonvulsant therapy may have no effect on levels of vitamin B12 [11]. In our patient, resolution was achieved without valproate discontinuation; this rules out its role. Possible causes of vitamin B12 deficiency in childhood include decreased intake, abnormal absorption and defects in vitamin B12 transport and metabolism [12]. As the Schilling test was not available at our center, we did the small bowel series which showed terminal ileum involvement. Had we not found any signs in her barium examination, we would have focused on other causes such as celiac disease, atrophic gastritis and food cobalamin malabsorption and performed the necessary tests. Based on our findings, we suspected that M. tuberculosis might be the cause of macrocytic anemia. As studies have shown, segmental involvement of the distal ileum, such as seen in tuberculosis, regional enteritis and Whipple's disease, can cause macrocytic anemia without any other manifestations of intestinal malabsorption such as steatorrhea [5,6]. Our diagnosis was supported by cleared signs of stricture in the terminal ileum after completion of the anti-tuberculosis treatment.

Vitamin B12 deficiency should be considered in patients with neurologic features such as paresthesia, sensory deficits, urinary incontinence, dysarthria, and ataxia. The underlying cause of B12 deficiency, which can include intestinal tuberculosis and other treatable causes, should be determined and treated to obviate the patient's need for long term vitamin B12 therapy.

## Abbreviations

MRI: Magnetic Resonance Imaging, LP: Lumbar puncture, EMG/NCV: Electromyography/Nerve Conduction Velocity, CBC: Complete Blood Count, MCV: Mean Corpuscular Volume

## Competing interests

The author(s) declare that they have no competing interests.

## Authors' contributions

TD carried out the patient management and diagnosis, and drafted the manuscript. FS participated in the patient management and the sequence alignment. AA participated in the sequence alignment. NR carried out the patient management and made the final diagnosis. MK participated in the manuscript design and coordination. All authors read and approved the final manuscript.

## Consent

Written informed consent was obtained from the patient for publication of this case report and any accompanying images. The patients' father also gave consent for the publication, as the patient is only 14 years old. A copy of the written consent is available for review by the Editor-in-Chief of this journal.

## Supplementary Material

Additional file 1 Table 1: CBC test. CBC test results show macrocytic anemia. Click here for file

Additional file 2 Table 2: Lab tests. All other blood tests are normal. Click here for file
