# Supplementary material for: Neuropathy caused by B12 deficiency in a patient with ileal tuberculosis: A case report
Source: J Med Case Rep. 2008 Mar 21;2:90. doi: 10.1186/1752-1947-2-90 (PMC2329654; doi:10.1186/1752-1947-2-90)
Supplement: Additional file 1 — Table 1: CBC test. CBC test results show macrocytic anemia. [file 1752-1947-2-90-S1.doc]

| **Table 1: CBC test** | | |
| --- | --- | --- |
| **Test** | **Result** | **Normal** |
| WBC | 7.7×10^3^ | 4-10 × 10^3^/ ul |
| RBC | 2.33×10^6^ | 4.1- 5.1 × 10^6^/ ul |
| HGB | 8.3 | 12- 15 gr/dl |
| HCT | 26.1 | 35- 45 % |
| MCV | 112 | 80- 100 fl |
| MCH | 35.6 | 27- 33 pg |
| MCHC | 35.7 | 31- 36 gr/dl |
| PLT | 197× 10^3^ | 150- 450 × 10^3^ /ul |
| ESR | 71 |  |
| Reticulocyte count | 0.9% |  |
