# Supplementary material for: Neuropathy caused by B12 deficiency in a patient with ileal tuberculosis: A case report
Source: J Med Case Rep. 2008 Mar 21;2:90. doi: 10.1186/1752-1947-2-90 (PMC2329654; doi:10.1186/1752-1947-2-90)
Supplement: Additional file 2 — Table 2: Lab tests. All other blood tests are normal. [file 1752-1947-2-90-S2.doc]

| **Table 2: Lab tests** | | | | | |
| --- | --- | --- | --- | --- | --- |
| **Test** | **Result** | **Normal** | **Test** | **Result** | **Normal** |
| PT | 15.7 | Control= 11 Sec | FANA | < 1/40 | < 1/ 40 |
| INR | 1.5 |  | Anticardiolipin total | 6 | positive> 10 |
| PTT | 41 | 25- 43 Sec | Serum ceruloplasmin | 36.2 | 23.6- 47 |
| Sodium | 144 | 135- 145 meq/ L | Antiphospholipid Ab (IgG) | 7.0 |  |
| Potassium | 3.6 | 3.5- 5 meq/ L | P- ANCA | < 5 | n< 5 |
| RF | Negative |  | C- ANCA | < 5 | N< 5 |
| ANA | <1 | >1 positive | RPR | nonreactive |  |
| Bill- total | 0.63 | 0.1- 1 mg/ dl | PPD | negative |  |
| Bill- Direct | 0.12 | < 0.3 mg/ dl | T4 | 9.8 | 4.8 - 13 |
| AST | 34 | 5- 40 IU/ L | T3 | 1.6 | 0.8- 2.1 |
| ALT | 40 | 5- 40 IU/ L | T3RU | 27% | 25- 35 |
| ALP | 142 | 64- 306 IU/ L | FTI | 2.75 | 1.1- 4.7 |
| Urea | 8 | 10- 50 mg/ dl | TSH | 4.2 | 0.3- 7.1 |
| Creatinine | 0.6 | 0.6- 1.3 mg/ dl | Stool exam | normal |  |
| LDH | 509 | 225- 500 IU/ L | CRP | +++ |  |
| CK | 166 | 24- 170 IU/ L | Albumin | 3.9 | 3.5- 5.5 gr/ dl |
| FBS | 105 | 45- 130 mg/ dl | Mg | 2.03 | 1.9- 2.5 mg/ dl |
| Urine analysis | Normal |  | Phosphorus | 4.9 | 2.5- 5 mg/ dl |
| HIV mix test | negative |  | Calcium | 8.9 | 8.6- 10.3 mg/dl |
| Fe | 78 | 40- 55 micg/ dl | Coombs Wright | Negative |  |
| TIBC | 220 | 200- 400 micg/ dl | Wright | Negative |  |
| Ferritin | 257.4 | 10- 160 micg/ dl | Serum folic acid | 17 | 1.5-16.5 ng/ml |
